# A qualitative focus group study concerning perceptions and experiences of Nigerian mothers on stillbirths

**DOI:** 10.1186/s12884-021-04207-4

**Published:** 2021-12-14

**Authors:** R. Milton, F. I. Alkali, F. Modibbo, J. Sanders, A. S. Mukaddas, A. Kassim, F. H. Sa’ad, F. M. Tukur, B. Pell, K. Hood, P. Ghazal, K. C. Iregbu

**Affiliations:** 1 Centre for Trials Research, Cardiff University, Cardiff, UK; 2 Department of Biochemistry, Bayero University, Kano, Nigeria; 3 Murtala Muhammad Specialist Hospital, Kano, Nigeria; 4 School of Healthcare Sciences, Cardiff University, Cardiff, UK; 5 Department of Biological Sciences, Bayero University, Kano, Nigeria; 6 Department of Medical Microbiology and Parasitology, Bayero University, Kano, Nigeria; 7 Centre for the Development and Evaluation of Complex Intervention for Public Health Improvement, Cardiff University, Cardiff, UK; 8 Systems Immunity Research Institute, School of Medicine, Cardiff University, Cardiff, UK; 9 Department of Medical Microbiology, National Hospital, Abuja, Nigeria

**Keywords:** Stillbirth, Stillborn, Sub-Saharan Africa, Nigeria, Northern Nigeria, Female, Mothers, Healthcare, Preventable mortality, Experiences, Cultural, Thematic analysis, Focus groups

## Abstract

**Objective:**

To explore the experiences and perceptions of stillbirth among mothers from a tertiary medical centre in Kano, Northern Nigeria.

**Design:**

Qualitative, interpretative.

**Setting:**

Tertiary healthcare facility, Murtala Muhammad Specialist Hospital (MMSH), Kano, Northern Nigeria.

**Sample:**

Mothers who had given birth to a liveborn baby at the MMSH in the prior 6 months (*n* = 31). In order to capture the experiences and perception of stillbirth within this cohort we approached mothers who had in a previous pregnancy experienced a stillbirth. Of the 31 who attended 16 had a previous stillbirth.

**Methods:**

Semi-structured Focus Group Discussions, consisting of open-ended questions about stillbirth, beliefs, experiences and influences were held in MMSH, conducted over 1 day.

**Results:**

Our findings highlight that this is a resource-poor tertiary facility serving an ever-growing population, increasing strain on the hospital and healthcare workers. Many of the participants highlighted needing permission from certain family members before accessing healthcare or medical treatment. We identified that mothers generally have knowledge on self-care during pregnancy, yet certain societal factors prevented that from being their priority. Judgement and blame was a common theme, yet a complex area entwined with traditions, superstitions and the pressure to procreate with many mothers described being made to feel useless and worthless if they did not birth a live baby.

**Conclusions:**

As access to healthcare becomes easier, there are certain traditions, family and social dynamics and beliefs which conflict with scientific knowledge and act as a major barrier to uptake of healthcare services. The findings highlight the need for investment in maternity care, appropriate health education and public enlightenment; they will help inform appropriate interventions aimed at reducing stigma around stillbirth and aide in educating mothers about the importance of appropriate health seeking behaviour. Stillbirths are occurring in this area of the world unnecessarily, globally there has been extensive research conducted on stillbirth prevention. This research has highlighted some of the areas which can be tackled by modifying existing successful interventions to work towards reducing preventable stillbirths.

**Supplementary Information:**

The online version contains supplementary material available at 10.1186/s12884-021-04207-4.

## Background

Globally, mothers in Sub-Saharan Africa (SSA) and South Asia bear the greatest burden of stillbirth. In 2019, over 75% of estimated global stillbirths occurred across these two continents: 44% in SSA and 33% in South Asia [1]. UNICEF report that the estimated stillbirth rate in SSA is 21.7/1000 total births; seven times higher than the lowest regional average stillbirth rate of 2.9/1000 births found in Western Europe [1]. Nearly all, 98%, of the world’s stillbirths occur in low- and middle-income countries (LMICs) and it is reported that Nigeria accounts for 12% (*n* = 312,000) of those [2]. For around half of stillbirths, intrauterine fetal death occurs during the intrapartum period [3, 4].

The neonatal mortality rate in Nigeria is 35.9/1000 live births, placing it as the sixth largest contributor to neonatal mortality after, Lesotho, Pakistan, South Sudan, Somalia and Afghanistan; predominantly countries experiencing severe conflict [5]. Stillbirth rates are more challenging to quantify and vary depending on data source. There are many reasons for the reported disparity including varying definitions of stillbirth, unreliable reporting systems, homebirths or unrecorded healthcare professional monitoring. It is therefore reasonable to assume that the reported rates are an underestimation [6].

Preliminary work carried out at the Murtala Muhammad Specialist Hospital (MMSH), Kano, found the incidence of stillbirth to be 180/1000 births (Milton et al, in press)*.* This statistic is significantly higher than any previously published finding. However, statistics do not represent the trauma of mothers who experience stillbirth, the trepidation throughout pregnancy and labour along with the physical and psychological sequalae. A number of risk factors are known to be associated with stillbirth namely, maternal and fetal infections, pre-eclampsia/eclampsia, asphyxia, obstructed labour and uterine rupture, fetal distress and umbilical cord complications, fetal growth restriction, placental abruption/placenta praevia [7, 8]. There is substantial evidence highlighting the psychosocial burden of stillbirth and following a renewed global focus on stillbirth as a global public health challenge this awareness has been heightened [9]. Religion and faith during extreme grief such as experiencing a stillbirth can be challenged and the belief structure questioned, conversely they can be a source of comfort or lead to increased reliance as a coping mechanism [9].

The unmet needs of mothers following stillbirth is well researched; many of the findings can also be applied to perceptions of stillbirth. Within communities it is common that women seeking support through community and family are often met with stigma, resulting in self-blame, isolation and ostracization, making them unable to grieve for the death of their baby [10, 11].

Other studies carried out in LMICs have identified associations between poor access to healthcare, decision-making dynamics within families on accessing healthcare, cultural beliefs, relationships between healthcare workers and mothers and stillbirth [12–14]. There is however, limited insight into lived experiences and perceptions of mothers in Kano. Only one study was identified that reported findings on the sociocultural understanding of miscarriages, stillbirths, and infant loss following interviews conducted with 35 Nigerian women [11]. Therefore, deepening this understanding is essential in order to address this public health concern.

This qualitative work was conducted as a component of ‘A feasibility study: Stillbirths in Kano’, the primary objective being to identify whether stillborn babies in MMSH can be classified using an established system. This larger study included qualitative, clinical epidemiology, microbiology and immunology components. An output of the feasibility study is to inform further research and pave the way for future intervention implementation to prevent stillbirth using a mixed-methods approach. The quality and veracity of the interventions will be based on more complete data and a holistic understanding of the population surveyed.

This manuscript focuses solely on the qualitative aspect, the aim of which was to explore and gain an understanding of some of the experiences and perceptions of mothers who experienced stillbirth to inform future research.

## Methods

### Study setting

This study was carried out in Murtala Muhammad Specialist Hospital (MMSH), Kano, Nigeria. MMSH is a tertiary hospital with 33 maternity beds and 22 delivery cubicles, managing approximately 650 births per month. Kano is the second largest city in Nigeria, next to Lagos, with an estimated population of 4,103,000, yet the MMSH serves a much greater population due to being one the most affordable hospitals which provides a wide range of services. Kano is predominantly a Muslim state with Hausa as the dominant ethnic group. Polygamous unions are widely accepted in predominantly Muslim states.

### Study design

**Fig. 1:**
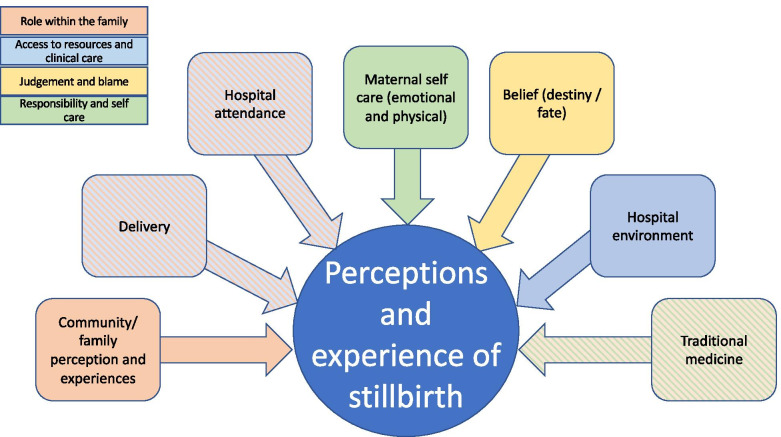
Coding structure: mothers’ perspectives of determinants of stillbirth in rural Nigeria; a visual demonstration of the coding structure the researchers used to categorise the findings in themes

A semi-structured interview guide consisting of open-ended questions about stillbirth, beliefs, experiences and influences was used to conduct Focus Group Discussions (FGDs) (Additional file; Fig. 1).

### Study participants

To capture different perspectives, two groups were formed: mothers with and without previous experience of stillbirth. Participants were divided into separate FGDs and were intended to be conducted independently, with the aim that the experience was as least stressful and upsetting as possible and we felt that mothers with shared lived experiences would be more open to discuss their experiences. The justification for including mothers who had not experienced stillbirth was to highlight any wider societal perceptions, traditions or beliefs which may not be captured from the mothers who had experienced stillbirth.

Our study sample were participants of the wider feasibility study who gave birth to a live baby at MMSH within the study period. Using data collected on previous pregnancy experiences, the researchers approached 30 mothers who had previously experienced a stillbirth and 30 mothers who had not. Women who had experienced stillbirth in their most recent pregnancy were excluded on the basis of sensitivity. Potential participants were contacted post-partum, over the telephone by a familiar study research nurse to provide them information on the FGDs and inviting them to participate. Forty women agreed to participate in the FGDs and 31 attended. The decision was made not to present maternal characteristics or demographic data to maintain anonymity and to increase participation in the FGDs.

### Procedures

Three FGDs (the third was late arrivals) were conducted over the course of 1 day (22/01/2019). Overall, three moderators (Doctor FZM; research nurses: ASM and AK) facilitated each session with the assistance of two note takers; for the late-comers two moderators and one note taker conducted the session. The FGDs were led by an experienced qualitative researcher (FZM), the other two moderators (ASM and AK) were moderating for the first time after training and support from the lead moderator. The sessions were conducted in a private room at the MMSH. Each FGD was audio recorded, lasting between 30 and 53 min without interference and conducted in Hausa, the local language. None of the researchers had any relationship with participants prior to study. Table 1 details the participants within the FGDs.

**Table 1:** Focus Groups; details the group number, the number of respondents within each group and the description of the group that each respondent participated in

**Group Number**	**Group Description**	**Number of Respondents**	**Duration in minutes**	**In text reference**
1	Mothers whose babies had all been live births.	11	35	RX FGD1
2	Respondents who had previously had a stillborn baby. However, not in their most recent pregnancy.	12	53	RX FGD2
3	Respondents who missed the appointment time and arrived late. This group was made up of four respondents who had never had a stillbirth (group 1) and four respondents who had previously had a stillbirth (group 2).	8	30	RX FGD3-1 RX FGD3-2

### Data analysis

FGDs were conducted in Hausa, the transcripts were later transcribed and translated into English by two bi-lingual research nurses (FIA and FHS) who were present at the FGDs. An inductive thematic analysis was adopted to identify patterned meaning across the FGDs [15]. Inductive thematic analysis was chosen to remain as close to the meanings in the grounded data as possible [16]. The FGDs aimed to capture real experiences of women who had been subjected to societal opinions directly or indirectly, as well as gaining an understanding of how these perceptions, behaviours and experiences influenced and impacted the decision and experience of the participant.

Following the steps of Braun and Clarke for thematic analysis the authors familiarised themselves with the data, reading and re-reading the transcripts, writing down initial thoughts [15]. Both then created initial coding frameworks separately; the first author (RM) using Nvivo 12 and the second author (FIA) coding manually using Microsoft Word. Interpretative analysis was carried out by using the collated codes, data extracts were categorised according to the overarching themes. Subsequently, identified themes were reviewed and analysed. At this stage some themes were refined, and some were combined due to significant similarities. Identified themes were defined and then named. The authors conducted blind coding and shared consistent findings with each other, some refinement of themes was required, and these aspects were discussed. The identified themes were ‘access to resources and clinical care’; ‘role within the family’, ‘responsibility and self-care’ and ‘judgement and blame’. Figure 1 shows the coding structure used.

## Results

### Access to resources and clinical care

Logistical access to resources and clinical care includes travel distance, availability of a suitable mode of transport or whether someone was available to take the mother to hospital. There are also less frequently discussed barriers to accessing healthcare, such as societal or familial perceptions and beliefs to accessing healthcare; within this section the focus remains on logistical access. An understanding of the importance of intrapartum care in reducing stillbirths is vital due to the high proportion of fetal death occurring during intrapartum stage. It is not uncommon for the labour ward to be full, with reports of mothers being turned away and giving birth on the hospital floor or in car parks. One respondent, with a known complicated pregnancy, described how she was unable to make it to the hospital in time to save her baby.

*This time I couldn't get to the hospital on time and it [stillbirth] was destined to happen. (R8 FGD2)*

Other respondents acknowledged that logistical access to the hospital is a high risk for stillbirth.

*Women that live in remote areas are at risk of stillbirth because they find it difficult to reach the hospital due to distance. (R8 FGD3-2)*

*I feel women living in remote areas are at risk of stillbirth because they find it difficult to reach the hospital due to bad roads and lack of vehicles. (R10 FGD1)*

Reports of lack of bedspace, waiting or being asked to come back were common.

*The first time I came, I was told that there was no bedspace in the labour room. However, after the nurse examined me, I was given a bed and I delivered my baby immediately. (R1 FGD1)*

Women often overcome several barriers to reach a hospital, only to give birth on the floor, unattended by a midwife, due to overcrowding and limited bedspace. A respondent shared that in a previous pregnancy she was unable to secure a hospital bed, and this resulted in a floor birth.

*In a previous pregnancy, I came to this hospital and was told that there was no bedspace. I gave birth on the floor. (R3 FGD1)*

The following respondent described being refused admission on initial presentation, which lead to a delay in admission and required caesarean section.

*The first time I came, I did not get admitted because there was no bedspace. However, when I came back the second time I got admitted, booked for c-section.... (R1 FGD2)*

There were examples of the hospital working above capacity with some women reporting that they were inadequately attended to in labour or until presentation of the fetal head. One respondent reported witnessing four women give birth unattended and suggested an association between lack of intrapartum care and stillbirth.

*I was told to go and lie down and try my best to deliver on my own when I complained to the midwife about pains. She only came when I alerted her that the baby's head was out. (R2 FGD1)*

*I had to alert the midwife that the baby’s head was out after which she came and conducted the delivery and cleaned up the baby. (R2 FGD2)*

*Four women delivered without the help of a midwife and they only came after the women had delivered by themselves. This kind of negligence can cause stillbirth. (R2 FGD3-1)*

A respondent reported in a previous pregnancy (outcome not stated) that she was unsupported during a complex breech birth.

*I gave birth on the floor; I was told that the baby was not lying correctly (breech) and was told by Doctor X not to deliver by myself, but I didn’t get admitted. (R1 FGD2)*

These narratives emphasise the fact that demands far outstrip available human resource capacities and infrastructure capabilities at the MMSH. Nevertheless, there were other respondents’ experiences that contrasted with some of the above, with reports that mothers were attended throughout birth and received attentive support and aftercare.

*The midwife was with me and dressed the baby after delivery. (R6 FGD1)*

*The midwife was by my bedside because I came in fully dilated. She conducted the delivery and cleaned me up before leaving my side. (R1 FGD1)*

Capacity on the postnatal wards was also an issue and although bedsharing during a hospital stay in Nigeria is common [17], babies are often also in the beds with mothers, which could lead to the safety of the babies being compromised as well as increasing the risk of postnatal and neonatal sepsis.

*We were overcrowded and had to stay two people per bed. (R9 FGD2)*

Many respondents detailed wholly positive birthing experiences, expressing gratitude to healthcare workers demonstrating compassion and understanding for the working environment and the stresses.

*After delivery, I was trying to clean the place up. The cleaning staff told me to stop doing it as cleaning is their job. She proceeded to clean the place. I thanked her and was very impressed. (R5 FGD2)*

*There was no bedspace, but the healthcare workers tried their best to get one for me and I was treated with good care. (R3 FGD2)*

Other respondents provided some examples of disrespectful interactions between healthcare workers and mothers.

*I have seen a patient turned away because she talked back to a midwife. She gave birth in the parking lot… (R10 FGD2)*

Two respondents described complications and altercations with midwives when intravenous cannula become displaced.

*A midwife was mad at me for removing the cannula that was set. I told her it was not intentional, but she did not believe me. She set a new line, but she kept complaining. (R6 FGD2)*

*A midwife kept yelling at me for removing my cannula (by mistake). She refused to put another one for a whole day. (R9 FGD2)*

One respondent described how the rapport with healthcare workers can contribute to the burden of labour.

*The midwives are not nice and are always shouting at patients. You cannot do anything to please them. They always seem angry. When in labour you have to deal with the pain and their attitude. (R8 FGD2)*

*The wrapper that my baby was covered in was wet. I asked the cleaning staff to help call my relative to clean the baby up and she declined. (R2 FGD1)*

The communication difficulties are expressed as a two-way challenge; with many mothers not listening to the midwives.

*Some patients refuse to listen to the midwife's instructions during delivery… and this causes stillbirth. I feel if women follow the midwife's instructions during delivery it will prevent stillbirth. (R5 FGD1)*

This theme details a broad spectrum of experiences with the outcome of the pregnancy likely to bias the perception of the experience. Access to healthcare can be challenging and complicated by the tradition that labour and birth are viewed as a female only affair independent of the husband [18]. Whilst a lack of respectful care on occasions caused distress to women, staff were working in a resource poor hospital, with inadequate bedspace and staffing levels to provide a basic level of intrapartum care to all women who attended the hospital.

### Role within the family

Within polygamous relationships an element of competition and comparison was observed.

*So far, I have had three (3) c-section with only one live born baby. Meanwhile the second wife has been giving birth safely by herself [SVD]. (R2 FGD2)*

Stillbirth is a traumatic experience, physically and emotionally; experiencing feelings of being replaceable or dispensable during medical or psychological care following such a devastating loss, exacerbates the trauma. The following account is an observed experience of one of the respondents who bedshared with another mother.

*The last baby was also stillborn and when she woke up after the surgery and asked the mother-in-law (who was by her bedside) about her baby, the mother-in-law said “Which baby? You gave birth to what you usually give birth to, a stillborn”. (R3 FGD2)*

Polygamous relationships enable women to learn from their co-wives and experiences in childbirth. This following account is a learned experience through fear, where the outcome discouraged home-birthing.

*I became afraid of delivery at home when my co-wife delivered her last baby. She had prolonged labour and was taken to another hospital where she was told that the baby was not lying correctly……and she delivered a stillborn…. (R4 FGD1)*

This following statement reflects how the household determine access to healthcare.

*I live in a traditional house with my husband’s relatives. No one goes to the hospital to give birth to a baby. Usually, deliveries are conducted at home by elderly women and/or women that have given birth before. Sometimes, a traditional birth attendant is called post-delivery. (R1 FGD1)*

This statement reflects the power dynamic within the family, the husband and family making decisions about childbirth. Participants talked about spousal participation during labour and birth. One respondent reported how her husband supports her accessing healthcare and will enable access. Another discussed how their husband ‘allows’ her to access healthcare, a contrast to the language from the respondent from FGD2 who used the term ‘encourages’.

*My husband does not agree with the practice of giving birth at home, so he allows me to go to the hospital. (R2 FGD1)*

*My husband encourages us to go to the hospital because he says it is very important. (R4 FGD2)*

*My husband is very supportive of me and if I say I am going to the hospital he does not stop me and will sometimes go out of his way to take me to the hospital. (R5 FGD2)*

However, some mothers experienced having restricted access to healthcare by family members and traditions, with encouragement to use homeopathic or traditional methods before accessing a hospital. A respondent detailed her experience of a prolonged labour and caesarean section and how she required her father to intervene to gain access to appropriate healthcare.

*My husband’s relatives told him that there are traditional medicines that will make me deliver my baby by myself. I took them and my water broke, and for three days I endured the pain, but nothing happened. I called my father and told him about the situation. He told my husband to take me to the hospital so that even if I would have given birth by myself [SVD] it should be in the hospital. The doctor said I cannot deliver by myself [SVD] and if I had stayed any longer at home, I would have had a ruptured uterus. I delivered via c-section. (R2 FGD2)*

A respondent described how the importance of accessing healthcare is discussed among peers.

*Those that have experience with delivery in the hospital educate them [friends/family] on the importance of delivery in the hospital as it pertains to complications like breech presentation and bleeding before and after delivery. (R6 FGD1)*

To summarise, the role of the mother within the family is directly linked to accessibility of correct healthcare and appropriate treatment.

### Responsibility and self-care

It is viewed as the primary responsibility of the mother to follow advice given in pregnancy including to; rest, eat healthily, avoid alcohol and cigarettes and seek appropriate healthcare. There were inferences that lack of self-care including not seeking appropriate healthcare, not seeking antenatal care, eating unhealthily or having heightened emotions could result in stillbirth.

*I feel that not going for antenatal and trying to keep count of the months of pregnancy by themselves leads to the mother not knowing that the baby has stayed too long in her womb. This could cause stillbirth. (R3 FGD3-2)*

*Women that do not go for antenatal sessions and [do not] take vaccines are at risk of convulsions during pregnancy and this can cause stillbirth and maternal death. (R11 FGD1)*

*I feel that lack of self-care is one of the causes of stillbirth. Some women do not eat good food that will be enough nourishment for them and the baby. (R1 FGD2)*

The term negligence was used, on the mother’s part through lack of appropriate care seeking behaviour.

*The mothers do not present early at the hospital. They wait until the labour has progressed and they have become too weak to push during delivery. This could cause stillbirth. (R2 FGD3-1)*

Many respondents believe poor emotional wellbeing is a contributor to stillbirth, one respondent shared the reasons she believed to be the cause.

*I feel strong emotions can cause stillbirth. Prior to delivering my stillborn, I had a falling out with my husband, and I wanted a divorce, but he was not cooperating…. I became depressed and refused to go for antenatal. I fell sick and had to be rushed to the hospital where the doctor told me that my blood pressure was too high….and delivered my baby by myself [SVD]. It was stillborn. If my emotions had been in check during the pregnancy, this could have been avoided. (R7 FGD3-2)*

The following respondent described the pressures of society on a mother-to-be.

*Lack of self-care is the major cause [of stillbirth]. Some women stress themselves with house chores. Some cannot be seen resting for fear of people calling them lazy. (R2 FGD2)*

Lack of antenatal care reduces the chances of complications being identified. For example, not having a confirmed due date can lead to an unrecognised post-term pregnancy, and regular monitoring of blood pressure is required to detect pre-eclampsia.

### Judgement and blame

Judgement and blame were found to have an important role in perceptions of stillbirth suggesting perhaps there is a deep-rooted need to find someone to blame for stillbirth. One respondent described a friend’s experience of being branded useless for giving birth to a stillborn baby.

*Her mother-in-law blamed her for giving birth to a stillborn and always tells her husband that she thinks his wife is not useful and wouldn’t give birth to children. (R3 FGD2)*

Mothers were exposed to feelings of disappointment within the husband’s family. One respondent described how she was mocked, and her husband was encouraged to marry another woman.

*They kept mocking me and encouraged my husband to get married to another woman that will give birth to live babies. (R1 FGD2)*

This finding was not unique, another respondent (FGD2) who had two previous stillbirths was aware her husband’s family encouraged him to find another wife.

*...so, they encouraged my husband to get married to another wife that will give birth to a live baby…. (R2 FGD2)*

Stillbirth is relatively common in Kano, and to an extent seemingly accepted in some cases. Many of the respondents stated that the birth outcome was the woman’s destiny or was meant to be.

*…for any woman that gives birth to a stillborn, it was destined to happen. (R1 FGD2)*

*Stillbirth is something that is destined for the woman that delivers a stillborn. (R2 FGD1)*

Regardless of the belief in destiny, there is a strong blame culture, that stillbirth is the fault of the mother. This respondent shares the feelings of her husband’s family following a stillbirth.

*After having a stillbirth, I took a long time before getting pregnant again. My husband’s relatives blamed me and said it does not seem as if I will give birth to another child. (R1 FGD2)*

Despite the findings in the responsibility and self-care theme a respondent from FGD2 described her experience of being mocked for attending hospital frequently and her family considering this as the cause of stillbirth.

*They [husband’s relatives] would mock me, saying I go to the hospital for the smallest sicknesses. When I gave birth to my first baby, it was stillborn. They blamed it on my frequent hospital visits. (R1 FGD2)*

Cultures develop over generations; superstitions occur and evolve and are engrained. Some respondents described humiliating and distressing treatment from their family and community following stillbirth.

Table 2 shows the comparison of some selected quotes from the FGDs.

**Table 2:** A comparison of selected quotes across FGDs; displays some example quotations from both FGDs side by side as a reference for a section within the discussion

**FGD1: No previous stillbirth**	**FGD2: Previous stillbirth**
**Theme: Access to resources and clinical care**	
***Transport***	
I feel women living in remote areas are at risk of stillbirth because they find it difficult to reach the hospital due to bad roads and lack of vehicles. (R10 FGD 1)	Women that live in remote areas are at risk of stillbirth because they find it difficult to reach the hospital due to distance. (R8 FGD3-2)
***Bedspace***	
The first time I came, I was told that there was no bedspace in the labour room. However, after the nurse examined me, I was given a bed and I delivered my baby immediately. (R1 FGD1)	The first time I came, I did not get admitted because there was no bedspace. However, when I came back the second time I got admitted, booked for c-section.... (R1 FGD2)
In a previous pregnancy, I came to this hospital and was told that there was no bedspace. I gave birth on the floor. (R3 FGD1)	There was no bedspace, but the healthcare workers tried their best to get one for me and I was treated with good care. (R3 FGD2)
	We were overcrowded and had to stay two people per bed. (R9 FGD2)
***Delivery***	
I was told to go and lie down and try my best to deliver on my own when I complained to the midwife about pains. She only came when I alerted her that the baby’s head was out. (R2 FGD1)	I had to alert the midwife that the baby’s head was out after which she came and conducted the delivery and cleaned up the baby. (R2 FGD2)
The midwife was by my bedside because I came in fully dilated. She conducted the delivery and cleaned me up before leaving my side. (R1 FGD1)	I gave birth on the floor; I was told that the baby was not lying correctly (breech) and was told by Doctor X not to deliver by myself, but I didn’t get admitted. (R1 FGD2)
***Aftercare***	
The midwife was with me and dressed the baby after delivery. (R6 FGD1)	After delivery, I was trying to clean the place up. The cleaning staff told me to stop doing it as cleaning is their job. She proceeded to clean the place. I thanked her and was very impressed. (R5 FGD2)
The wrapper that my baby was covered in was wet. I asked the cleaning staff to help call my relative to clean the baby up and she declined. (R2 FGD1)	
***Interactions***	
Some patients refuse to listen to the midwife’s instructions during delivery… and this causes stillbirth. I feel if women follow the midwife’s instructions during delivery it will prevent stillbirth. (R5 FGD1)	The midwives are not nice and are always shouting at patients. You cannot do anything to please them. They always seem angry. When in labour you have to deal with the pain and their attitude. (R8 FGD2)
**Theme: Role within the family**	
***Experiences within the household***	
I became afraid of delivery at home when my co-wife delivered her last baby. She had prolonged labour and was taken to another hospital where she was told that the baby was not lying correctly……and she delivered a stillborn…. (R4 FGD1)	So far, I have had three (3) c-section with only one live born baby. Meanwhile the second wife has been giving birth safely by herself [SVD]. (R2 FGD2)
	The last baby was also stillborn and when she woke up after the surgery and asked the mother-in-law (who was by her bedside) about her baby, the mother-in-law said “Which baby? You gave birth to what you usually give birth to, a stillborn”. (R3 FGD2)
***Accessing healthcare***	
I live in a traditional house with my husband’s relatives. No one goes to the hospital to give birth to a baby. Usually, deliveries are conducted at home by elderly women and/or women that have given birth before. Sometimes, a traditional birth attendant is called post-delivery. (R1 FGD1)	My husband’s relatives told him that there are traditional medicines that will make me deliver my baby by myself. I took them and my water broke, and for 3 days I endured the pain, but nothing happened. I called my father and told him about the situation. He told my husband to take me to the hospital so that even if I would have given birth by myself [SVD] it should be in the hospital. The doctor said I cannot deliver by myself [SVD] and if I had stayed any longer at home, I would have had a ruptured uterus. I delivered via c-section. (R2 FGD2)
My husband does not agree with the practice of giving birth at home, so he allows me to go to the hospital. (R2 FGD1)	My husband is very supportive of me and if I say I am going to the hospital he does not stop me and will sometimes go out of his way to take me to the hospital. (R5 FGD2)
	My husband encourages us to go to the hospital because he says it is very important. (R4 FGD2)
**Theme: Responsibility and self-care**	
Women that do not go for antenatal sessions and [do not] take vaccines are at risk of convulsions during pregnancy and this can cause stillbirth and maternal death. (R11 FGD1)	I feel that not going for antenatal and trying to keep count of the months of pregnancy by themselves leads to the mother not knowing that the baby has stayed too long in her womb. This could cause stillbirth. (R3 FGD3-2)
The mothers do not present early at the hospital. They wait until the labour has progressed and they have become too weak to push during delivery. This could cause stillbirth. (R2 FGD3-1)	I feel that lack of self-care is one of the causes of stillbirth. Some women do not eat good food that will be enough nourishment for them and the baby. (R1 FGD2)
**Theme: Judgement and blame**	
Stillbirth is something that is destined for the woman that delivers a stillborn. (R2 FGD1)	…for any woman that gives birth to a stillborn, it was destined to happen. (R1 FGD2)
	Her mother-in-law blamed her for giving birth to a stillborn and always tells her husband that she thinks his wife is not useful and wouldn’t give birth to children. (R3 FGD2)
	They kept mocking me and encouraged my husband to get married to another woman that will give birth to live babies. (R1 FGD2)
	They [husband’s relatives] would mock me, saying I go to the hospital for the smallest sicknesses. When I gave birth to my first baby, it was stillborn. They blamed it on my frequent hospital visits. (R1 FGD2)

## Discussion

### Summary of the main findings

Clear and important messages were identified from each theme. Although women are encouraged to attend hospital for birth, which may involve a long or difficult journey, the hospital was often found to have insufficient bedspace or staffing to provide basic intrapartum care to all women attending. The inability to provide care to all the women encouraged to attend on occasions resulted in unattended births or women giving birth in the carpark or on the floor.

Healthcare workers are over-stretched and under intense pressure. Reported interactions with healthcare workers included compliments from women grateful at the care provided to themselves and their babies, but also reports of negative verbal exchanges which may have been a reflection of the moral distress and burn out among the healthcare workers due to having to work in very challenging environments [19]. A significantly limited resource facility such as the MMSH having to manage an approximately 20-25 deliveries daily, with four core midwives per shift, has the potential of excessively exhausting the staff leading to less than optimal performance and output.

The role of the family in choosing the planned place of birth and responding to stillbirth is complex. Kano’s culture cannot be fully understood without considering the role of religion and ethnic characteristics; for example, a poor outcome such as stillbirths are often redefined as destiny [20]. Much as this may help to strengthen the woman emotionally, it often leaves the obvious causes unattended, giving rise to recurrence.

The influence of society in this setting is a tangled web of traditions, superstitions and the need to procreate. In SSA children are viewed as markers of prestige, and if a woman is unable to give birth to a live baby it is often perceived as a failing to her husband and his family. SSA is renowned for its pronatalist culture, with childless or infertile women classified as social deviants [21]. An added element of complication is that within polygynous marriages, it is reported that the husband allocates resources to wives based on the number of children they have [22]. As access to healthcare becomes more available certain traditions, family and social dynamics and beliefs can contradict scientific knowledge and act as a major barrier to uptake of services.

Participants demonstrated clear knowledge around the importance of self-care during pregnancy, yet this was compounded by societal pressure to continue with physical work for fear of judgement. Promoting self-care and encouraging this within local networks is vital. Benefits of accessing healthcare and antenatal care throughout pregnancy is being communicated through the communities, but the message could be spread more informatively and efficiently using other suitable media and fora. In Kano four antenatal check-ups are recommended after 16-weeks’ gestation. The midwives give health talks on the benefits of antenatal care, safety tips for the women to follow, hygiene advice and what to bring to the hospital for delivery. Physical check-ups, vitamins and vaccinations are also offered as well as one-to-one sessions with the midwife to discuss any concerns.

It was evidenced that judgement and blame for stillbirth stems from multiple sources, yet despite these blames, there is a strong belief and acceptance that destiny is central to all. Alluding to a greater interconnectivity of all themes, that a stillbirth must be “someone’s fault”, society blames mothers, mothers blame themselves or hospital staff and the cycle continues without seeking understanding around the possible causes.

### Comparisons and similarities between FGDs

Common perceptions across all participants were that those living more remotely were more likely to experience stillbirth due to the physical challenges of accessing healthcare, demonstrating that this is commonly experienced and an area for targeted intervention.

Mothers from both FGDs described the struggles of hospital bedspace, yet healthcare workers still tended to them and tried hard to obtain beds, even if it meant bedsharing, mothers from both FGDs described delivering on the floor. Mothers from both FGDs described having to call the midwife after presentation of the fetal head, a mother in FGD1 described how a midwife was present throughout delivery as she was fully dilated on presentation, this was not detailed by any mothers in FGD2, yet good and kind care was detailed in both FGDs. Highlighting unanimously that resources, physical and human are insufficient and that an investment in staff, equipment and beds is needed to improve the standard of clinical care required.

The interactions between mothers and healthcare workers were reflected interestingly; within FGD1 the mother described the patients (other mothers) refusing to listen to midwives and that the focus should be on listening to the healthcare professionals. Conversely in FGD2 a participant describes that the midwives are unkind. This poses the question of whether experience of interactions and treatment are reflective of outcome.

Contrasting experiences of living with co-wives were expressed between the FGDs; a mother from FGD2 detailed how her co-wife delivers live babies via SVD, yet for her she has had three Caesarean-sections and only one baby was a livebirth. A mother from FGD1 details how she had observed her co-wife suffer a complicated delivery resulting in stillbirth, and this had scared her, deterring her from delivering at home. Two different experiences resulting in different outcomes; but presenting the advantages and the disadvantages of shared lived experiences. Among participants of both FGDs there were examples of family members encouraging traditional medicine and birthing practices and also attending hospital for delivery, demonstrating the split in society around the use of traditional and modern medicine.

Perceptions that not attending ANC and lack of self-care were consistently reported by participants of both FGDs as causes of stillbirth, reflective of a good understanding of basic needs of an expectant mother. Participants of both FGDs believe that stillbirth is destined to happen, linking closely with the ability to seek comfort in faith and belief. Those with experience of stillbirth were able to share experiences of mockery or direct blame.

### Comparisons to previous literature

Antenatal care coverage has improved over recent years and at least one visit was recorded for 60.6% of Nigerian mothers in 2014 [23]. The existing WHO recommendation for antenatal clinic attendance is a minimum of four sessions; and between 2007 and 2014 only 64% of pregnant women attended four contacts [24]. However increasing from four to eight check-ups would be expected to significantly reduce stillbirth rates and MMSH is working towards implementing this.

Midwifery care has a pivotal role in the reduction of preventable maternal and neonatal mortality and morbidity [19]. Shakespeare conducted a systematic review looking at parents’ and healthcare professionals’ experiences of care after stillbirth in LMICs, healthcare workers reported lack of sufficient resources, including facilities, equipment, and staff shortages as barriers to providing good care [12]. These findings are reflected in the experiences of our FGD participants. This review highlighted barriers to accessing healthcare including the woman’s healthcare seeking decision-making process being domiciled with the husband or the mother-in-law.

It is now recognised that poor person-centred maternity care (PCMC), defined as disrespectful and neglectful treatment of women during facility deliveries, occurs in many countries regardless of income status [14]. Afulani identified that poor PCMC can be detrimental to women accessing healthcare and ultimately contribute to poor pregnancy outcomes [14]. The WHO are promoting the implementation of The Respectful Maternity Care Charter which has highlighted that disrespect and abuse of women seeking maternity care is a global problem requiring urgent attention [25]. The perceived cases of seeming disrespect in these FGDs cannot be taken out of context. It is difficult to distinguish actions or experiences occasioned by adverse work environment from acts of disrespect. It has been noted by patients that the facility is overcrowded with inadequate staff resources, who in turn may also be suffering work burn-out. This study perhaps suffers from the limitation of not having sought the views of the healthcare workers following the FGDs, something we hope to further explore in future research. To truly appreciate the situation and design the most appropriate mitigation all sides must contribute to the discussions.

Dahab found that the most common barriers to accessing healthcare are transportation, economic factors, and cultural beliefs, in addition to lack of family support and poor quality of care. All these featured in the findings of our FGDs [13].

### Strengths and weaknesses

A strength was that we were able to capture experiences of women who had previously experienced a stillbirth. Our concern while planning the FGDs was that women would not be willing to participate. We chose to split the mothers, out of respect and cautionary measure, but by default a mixed group evolved, which provided a different dynamic. In hindsight, separating the mothers based on their experiences was an overcautious decision which could have limited opportunities and discussions.

This research complements the wider feasibility study. This work provides context unable to be achieved through clinical epidemiological data and is a unique and informative piece of work, providing more information about an important topic where there is limited knowledge in this geographical location.

A limitation was that we only held FGDs with mothers; to have had the opportunity to obtain further insight into the situation and the perspectives of healthcare workers, fathers, grandmothers/mothers-in-law and other members of the community would have enabled the study to reflect a broader understanding of perceptions and experiences of stillbirth within Kano and provided a more complete story. This is an important area which future work can focus on.

### Implications

Stillbirth rates can be effectively reduced by high quality midwifery and maternity care, and the stillbirth rate in many parts of the world with similar income status and geographical similarities are exponentially lower than that in Kano. A logical next step would be to conduct or build on an existing systematic review of the successful stillbirth interventions in similar settings and to try to modify or implement existing interventions. It was reported that comprehensive obstetric services at delivery in LMICs would achieve the greatest stillbirth reduction worldwide; an estimated 700,000) [26]. Notwithstanding, governments in LMICs are financially constrained amid competing needs. For a state like Kano with a large population the support of individuals, groups, non-governmental organisation, local and international organisations will be required to substantially address the issue of high rate of stillbirth. More health centres are needed nearer to the people to ensure quick access and reduce the pressure at MMSH.

Self-blame and guilt are extremely detrimental to the mother following a stillbirth. Grief and depressive symptoms are a common experience, which could persist for years and prevent the mother to function normally [27]. Disenfranchised grief is often reported in connection with stillbirth, and the implications cause isolation and long-term distress to mothers following stillbirth [10, 11]. Disenfranchised grief is reported to be driven by the stigma surrounding stillbirth, for example not being able to see or name the baby and in some instances not being able to talk about the baby among family and friends [27]. Mother and family postnatal and bereavement support may be one such way to minimise the harm caused by the implications of self-blame, guilt and disenfranchised grief.

It is evident that the whole family are important in making informed decisions about planned place of birth and antenatal care. To reduce inequalities in antenatal and intrapartum care there needs to be an improvement in clinical resources and practice, combined with education aimed at changing behaviour and attitude at a societal level.

These data are a positive step in reducing stigma, in people talking about stillbirth and their experiences and perceptions. We hope that this review will encourage others to share their experience for others to learn and make informed decisions on their own healthcare.

### Author reflections

From the perspective of the first author, some of these scenarios and statements are worrisome and of concern. Living and working in the United Kingdom, with personal experience of childbirth being a well monitored, well supported and a clinician-led experience it is astonishing how diverse the situation and experiences could be between high-income (HICs) and LMICs could be. Having visited the MMSH and observing the overflowing units, seeing mothers two per bed, it does generate a reflective perspective of gratitude and highlights the privileges people have access to in HICs well as a focus on what is deemed acceptable. In terms of what was reported to be a lack of compassion shown to mothers’ who had delivered stillborn babies, some of the experiences appear strange, given that most communities in Nigeria live communal lives of support for family members. I am certain that despite best efforts reflexivity influenced how I interpreted the findings; yet working closely with the co-authors from Kano and other parts of Nigeria and drawing on work experience, retaining focus on the purpose of this research has enabled me to present the findings objectively and in transparent way.

## Conclusions

To assess a culture or a society with no lived experience seems simple, the flaws appear obvious and easy to correct, yet this is not reality; the complexities are deep rooted and require intelligent and well understood approaches to implement successful change. Throughout all the identified themes the pressures from society and culture were present, many messages seem conflicting; with varying degrees of acceptance of this common practice of blaming the mother for the outcome and disrespect to women who are unable to deliver live babies. Globally, mixed messages on societal perceptions are not uncommon, yet it’s important that messages surrounding societal perception aren’t more prominent than those surrounding the care of the mother and her baby.

There is an overarching and positive message conveyed throughout the FGDs; change is happening, positive messages about positive health seeking behaviour are being shared across networks and down through generations. The former Emir of Kano, Muhammed Sanusi II had, throughout his reign held strong views on the social justice of women, polygamy, and child marriage. The need to have the message disseminated faster and wider is integral to reducing the preventative stillbirth rate in Kano.

Many stillbirths are preventable with good antenatal and intrapartum care; for example identifying and monitoring high risk pregnancies and births [8, 28]. Investment in services, staff and resources combined with respectful care and education is now required to maximise improvements in perinatal outcomes in this region. A reasonable recommendation for intervention within this setting would be to screen mothers during their ANC visits and monitor high risk pregnancies throughout the course of their pregnancy. To improve the veracity and quantity of information available within Kano it would be pertinent to detail the conditions and associated pregnancy outcomes with immediate effect. These data would help inform the ANC team and subsequently hospital team as to the appropriate care required during birth.

## Supplementary Information

**Additional file 1: Figure 1.** Semi-structured interview guide.

## Data Availability

The transcripts analysed during this study are not publicly available, due this not forming part of our consent process, but are available from the corresponding author on reasonable request.

## References

[1] UNICEF. Stillbirths. Available from: https://data.unicef.org/topic/child-survival/stillbirths/. Cited 2021 Feb 8.

[2] Okonofua FE, Ntoimo LFC, Ogu R, Galadanci H, Mohammed G, Adetoye D (2019). Prevalence and determinants of stillbirth in Nigerian referral hospitals: a multicentre study. BMC Pregnancy Childbirth.

[3] WHO. Maternal, newborn, child and adolescent health. Available from: https://www.who.int/maternal_child_adolescent/epidemiology/stillbirth/en/. Cited 2019 Nov 7.

[4] Frøen JF, Gordijn SJ, Abdel-Aleem H, Bergsjø P, Betran A, Duke CW (2009). Making stillbirths count, making numbers talk - issues in data collection for stillbirths. BMC Pregnancy Childbirth.

[5] UNICEF. Neonatal mortality. Available from: https://data.unicef.org/topic/child-survival/neonatal-mortality/. Accessed Feb 2021.

[6] Lakew D, Tesfaye D, Mekonnen H (2017). Determinants of stillbirth among women deliveries at Amhara region, Ethiopia. BMC Pregnancy Childbirth.

[7] Aminu M, Bar-Zeev S, White S, Mathai M, Van Den Broek N (2019). Understanding cause of stillbirth: a prospective observational multi-country study from sub-Saharan Africa. BMC Pregnancy Childbirth.

[8] Goldenberg RL, Griffin JB, Kamath-Rayne BD, Harrison M, Rouse DJ, Moran K (2018). Clinical interventions to reduce stillbirths in sub-Saharan Africa: a mathematical model to estimate the potential reduction of stillbirths associated with specific obstetric conditions. BJOG.

[9] Nuzum D, Meaney S, O’Donoghue K (2017). The spiritual and theological challenges of stillbirth for bereaved parents. J Relig Health.

[10] Mills TA, Ayebare E, Mukhwana R, Mweteise J, Nabisere A, Nendela A (2021). Parents’ experiences of care and support after stillbirth in rural and urban maternity facilities: a qualitative study in Kenya and Uganda. BJOG.

[11] Adebayo A, Liu M, Cheah W (2019). Sociocultural understanding of miscarriages, stillbirths, and infant loss: a study of Nigerian women. J Intercult Commun Res.

[12] Shakespeare C, Merriel A, Bakhbakhi D, Baneszova R, Barnard K, Lynch M (2019). Parents’ and healthcare professionals’ experiences of care after stillbirth in low- and middle-income countries: a systematic review and meta-summary. BJOG.

[13] Dahab R, Sakellariou D (2020). Barriers to accessing maternal care in low income countries in Africa: a systematic review. Int J Environ Res Public Health.

[14] Afulani PA, Phillips B, Aborigo RA, Moyer CA (2019). Person-centred maternity care in low-income and middle-income countries: analysis of data from Kenya, Ghana, and India. Lancet Glob Health.

[15] Braun V, Clarke V (2006). Using thematic analysis in psychology. Qual Res Psychol.

[16] Smith J. Qualitative psychology: a practical guide to research methods. 2nd ed. London: SAGE Publications Ltd; 2007. p. 288.

[17] Bohren MA, Titiloye MA, Kyaddondo D, Hunter EC, Oladapo OT, Tunçalp Ö (2017). Defining quality of care during childbirth from the perspectives of Nigerian and Ugandan women: a qualitative study. Int J Gynecol Obstet.

[18] Vehviläinen-Julkunen K, Emelonye AU. Spousal participation in labor and delivery in Nigeria. Ann Med Health Sci Res. 2014;4(4):511–5 Available from: https://www.ajol.info/index.php/amhsr/article/viewFile/112389/102141%0Ahttps://www.ajol.info/index.php/amhsr/article/view/112389. 10.4103/2141-9248.139290 PMC4160671 25221695

[19] Filby A, McConville F, Portela A (2016). What prevents quality midwifery care? A systematic mapping of barriers in low and middle income countries from the provider perspective. PLoS One.

[20] Galanti G-A. Caring for patients from different cultures. 5th ed: Penn Press; 2014. p. 384.

[21] June N, Dimka R, Dein SL, Women’s Health and Action Research Centre (WHARC) (2018). The work of a woman is to give birth to children: cultural constructions of infertility in Nigeria. Afr J Reprod Health.

[22] Munro A, Kebede B, Tarazona M (2010). The lion’s share. An experimental analysis of polygamy in Northern Nigeria.

[23] Office of the Senior Specialist Assistant to the President on Millennium Development Goals (OSSAP-MDGs) (2015). Nigeria 2015 millennium development goals end-point report.

[24] WHO (2016). WHO Recommendations on antenatal care for a positive pregnancy experience.

[25] Fawcus S (2016). Respectful maternity care. Obstet Gynaecol Forum.

[26] Bhutta ZA, Yakoob MY, Lawn JE, Rizvi A, Friberg IK, Weissman E (2011). Stillbirths: what difference can we make and at what cost?. Lancet.

[27] Burden C, Bradley S, Storey C, Ellis A, Heazell AEP, Downe S (2016). From grief, guilt pain and stigma to hope and pride - a systematic review and meta-analysis of mixed-method research of the psychosocial impact of stillbirth. BMC Pregnancy Childbirth.

[28] Page JM, Silver RM (2017). Interventions to prevent stillbirth. Semin Fetal Neonatal Med.

