# Supplementary material for: A qualitative focus group study concerning perceptions and experiences of Nigerian mothers on stillbirths
Source: BMC Pregnancy Childbirth. 2021 Dec 14;21:830. doi: 10.1186/s12884-021-04207-4 (PMC8670111; doi:10.1186/s12884-021-04207-4)
Supplement: Supplementary file 1 — Additional file 1: Figure 1. Semi-structured interview guide. [file 12884_2021_4207_MOESM1_ESM.pdf]

Focus groups in Nigeria for the stillbirth study

- Two focus groups
- Location: to be held in MMSH with food / drink and a stipend for transport provided
- To be conducted by Zahra and two other researchers
- A minimum of 10 participants per focus group
- Recorded and transcribed.
- Conducted in local language – translation required.
- Duration: One hour

Number of Participants:

Date:

Moderator:

Note taker:

Start time:

End Time:

Focus Group Introduction

Purpose: The moderator greets the participants and explains the objectives of FGD. Setting the rules. Participants meet each other. Everyone says something, contributing to social facilitation.

Thank you for participating in this discussion group. This study is the first of its kind in this area, looking at the rates and risk factors of stillbirths. We would like to ask you all to join in a discussion about this topic and appreciate your thoughts and honesty on this subject. This is a very distressing subject matter and we really do appreciate you taking the time to contribute to this valuable research. We encourage you all to share your opinions and thoughts and also appreciate that we respect each other's thoughts and give everyone the opportunity to talk freely.

Discussion Guidelines

- We would like the discussion to be informal, so please feel free to express your views, by speaking openly and freely. It is also important that everyone speaks up, one at a time and not interrupt when someone else is speaking even if you disagree with what the person has to say, as you will have your turn to have

your say. It may also be necessary for the moderator to interrupt the discussion at certain times to ensure that everyone's opinions are included and all the topics are covered.

Confidentiality

- We will be audio tape recording the discussion so that we don't miss any of your comments. A report will be prepared after this, but participants names will NOT be used in any analysis of the discussion. Any information you provide will be strictly confidential. You will also be required to sign a consent form before we start. Please feel free to ask any questions you may have at this point.

Participant introductions

[MODERATOR SHOULD BEGIN, WITH A VERY BRIEF REFERENCE TO OWN PROFESSION AND FAMILY]

Let's go around the room - tell us something about yourself - first name only. Just say one thing about yourself, like where you were born. Just so we know enough about each other to have a good conversation. This part of our conversation will not be taped

FOCUS GROUP 1 - women who are enrolled on the study but have never had a stillbirth

Question 1: You all attended MMSH to deliver your babies, what were your main reasons for choosing to deliver in hospital? Can you tell us why you chose MMSH over other facilities in the area?

Probe: Is this your first hospital delivery? Did your husband insist? Is MMSH closer to your home? Is MMSH more affordable?

Question 2: Can you talk about your experience of delivering your baby at MMSH?

Probe: Did you experience any problems in pregnancy or delivery? Did you have a midwife present throughout delivery / labour? Any problems after delivery? How long did you stay in hospital?

Question 3: Were there any barriers to you attending hospital to delivery your baby?

Probe: Physical (transport) and social barriers, husband / family member not wanting you to go?

Question 4: Stillbirths are sadly common in this area, from your knowledge and experiences can you talk about your opinion on stillbirths and the potential risks and causes of stillbirths and whether you feel they could be prevented? If you feel they could be prevented could you share how?

Probe: what do you think are the reasons women have stillbirths?

Exclusion criteria

Focus Group 1:

- Women who have had a stillbirth.
- Nulliparous women.

FOCUS GROUP 2 - women who are enrolled on the study but have had a previous stillbirth (exclusion must be made for women who have had a stillbirth in this study).

Question 1: You all attended MMSH to deliver your babies, what were your main reasons for choosing to deliver in hospital? Can you tell us why you chose MMSH over other facilities in the area?

Probe: Is this your first hospital delivery? Did your husband insist? Is MMSH closer to your home? Is MMSH more affordable?

Question 2: Can you talk about your experience of delivering your baby at MMSH?

Probe: Did you experience any problems in pregnancy or delivery? Did you have a midwife present throughout delivery / labour? Any problems after delivery? How long did you stay in hospital?

Question 3: Were there any barriers to you attending hospital to delivery your baby?

Probe physical (transport) and social barriers, husband / family member not wanting you to go?

Question 4: Stillbirths are sadly common in this area, from your knowledge and experiences can you talk about your opinion on stillbirths and the potential risks and causes of stillbirths and whether you feel they could be prevented? If you feel they could be prevented could you share how?

Probe: what do you think are the reasons women have stillbirths?

Question 5: If you're comfortable discussing this, can you please talk about your previous stillbirth experience. Did you deliver in hospital? Did you experience any birthing complications?

Probe: How long did it take to get to MMSH, how did you travel? Did you have to wait for your husband to come home? How long had your waters broken / gone into labour before coming to hospital?

Exclusion criteria

Focus Group 2:

- Women who have not had a stillbirth.
- Women who have had a stillbirth in this study.
- Nulliparous women.
